# Emergency surgery for blunt abdominal trauma in polytrauma – the impact of time to laparotomy on outcomes

**DOI:** 10.1007/s00068-026-03332-z

**Published:** 2026-10-19

**Authors:** Gianmaria Rausch, Yannik Kalbas, Sascha Halvachizadeh, Jonin Zumsteg, Alba Shehu, Ester Mikova, Kuno Lehmann, Ladislav Mica, Roman Pfeifer, Hans-Christoph Pape, Valentin Neuhaus, Felix Karl-Ludwig Klingebiel, Michel Paul Johan Teuben

**Affiliations:** 1 Department of Trauma Surgery, University Hospital Zurich, University of Zurich, Ramistr. 100, Zurich, 8091 Switzerland; 2 Harald-Tscherne Laboratory for Orthopaedic and Trauma Research, University Hospital Zurich, University of Zurich, Ramistr. 100, Zurich, 8091 Switzerland; 3 North Bristol NHS Trust, Southmead Hospital, Bristol, UK; 4 Department of Surgery, Solothurn Hospital, Solothurn, Switzerland

**Keywords:** Blunt abdominal trauma, Laparotomy, Time to surgery, Emergency surgery, Polytrauma

## Abstract

**Purpose:**

Blunt abdominal trauma (BAT) is common in polytrauma patients. Emergency surgery is required for haemodynamically significant solid organ injury and intra-abdominal hollow organ injury. The effect of time to surgery on outcomes in patients requiring emergency laparotomy remains uncertain.

**Materials & methods:**

Adult polytrauma patients (ISS > 16) who underwent emergency laparotomy for blunt abdominal trauma between 1996 and 2020 from a trauma registry of a single level one trauma centre were included. Patients were divided into two groups based on the time elapsed from admission to emergency laparotomy: “Super Early” (≤ 60 min) and “Early” (60–180 min). Length of stay (LOS), intensive care unit (ICU)-LOS, ventilation time, re-look operations and mortality were assessed. Adjusted logistic regression was used to determine the independent predictive parameters, including timing of surgery, for mortality.

**Results:**

The study included 190 polytrauma patients with concomitant blunt abdominal trauma (mean age 42 years (± 17), median ISS of 36 (IQR: 19)). There were 95 patients in each group. Patients in the “Super Early” group had a compromised haemodynamic status and a higher ISS. Liver injury and serous membrane injury were more common in the super early group. The mortality rate was significantly higher in the “Super Early” group (51.6%) compared to the “Early” group (26.3%) (*p* < 0.001). Death due to haemorrhagic shock was significantly more frequent in the “Super Early” group (36.8%) compared to the “Early” group (13.7%) (*p* < 0.001). A regression model was adjusted to determine the independent predictors of mortality. Time to emergency laparotomy was not an independent predictor of mortality. However, increased ISS (OR = 1.04, *p* = 0.049), age (OR = 1.03, *p* = 0.037) and decreased systolic blood pressure (OR = 0.98, *p* = 0.025) were found to be significant predictors of mortality.

**Conclusion:**

The timing of surgery showed no independent association with mortality as long as an emergency laparotomy was performed within 3 h of admission. The data suggests that patient-specific characteristics, injury severity and clinical status are more important for survival than the timing of an early emergency laparotomy. Future research should focus on determining the role of delayed laparotomy on outcomes in specific subgroups of patients.

**Supplementary Information:**

The online version contains supplementary material available at https://doi.org/10.1007/s00068-026-03332-z.

## Introduction

Blunt abdominal trauma (BAT) represents a common injury pattern, accounting for up to 80% of all abdominal injuries observed in emergency rooms [1]. ***(Correction 1.8)*** The most commonly injured abdominal organs are the spleen and liver, followed by the hollow viscera and kidneys [2]. In cases of BAT in haemodynamically stable patients without hollow organ injury non-operative management is preferred [3]. Nevertheless, in certain cases, an emergency exploratory laparotomy is unavoidable. The indications for an emergency laparotomy are well established; however, the impact of its timing remains a topic of debate [4].

According to international guidelines, diagnostics in the trauma bay should preferably include either radiography and ultrasound, or whole-body computer tomography (CT) [5]. The goal is to achieve prompt identification of injuries, allowing for targeted intervention, while avoiding unnecessary surgical exploration [6–8]. Non-negligibly, these emergency diagnostics take time at a crucial stage.

Several studies have investigated the effect of time to surgery on patient outcome in penetrating abdominal trauma. Hershkovitz et al. found that in haemodynamically compensated patients with penetrating abdominal trauma, the timing of surgery, up to two hours after admission, did not affect outcome [9]. Similar results have been reported for isolated blunt abdominal trauma [10]. On the other hand, a study of a larger and more diverse population with severe polytrauma suggests that prompt surgical intervention may prevent early trauma deaths [11].

The aim of this study is to determine the impact of time to emergency surgery for BAT in polytraumatized patients.

*It was hypothesised that “super early” emergency laparotomy (< 60 min after admission) is associated with a better outcome than “early” emergency laparotomy (60–180 min) in polytrauma patients with BAT.*

## Materials and methods

### Setting

The study was conducted in accordance with the Declaration of Helsinki [12] and the STROBE guidelines (STROBE: Strengthening the Reporting of Observational Studies in Epidemiology). Ethical approval was obtained from the Swiss Cantonal Ethics Committee (BASEC No. 2021 − 00391).

### Study type

This is a retrospective cohort study of polytrauma patients who underwent emergency laparotomy for BAT at the University Hospital of Zurich between 1996 and 2020.

### Study population

The data used were extracted from the polytrauma database of the University Hospital of Zurich, a certified Swiss level 1 trauma centre. This trauma registry includes all patients who were admitted to our trauma bays between 1996 and 2020.

Inclusion criteria: Blunt abdominal trauma, ISS > 16, laparotomy and time to emergency surgery < 3 h.

Exclusion criteria: Age < 16 years, external referrals and AIS head > 4.

### Study conditions

Patients were categorised into two groups based on the time elapsed between admission to the trauma bay and the time of incision for emergency laparotomy. ***(Correction 1.2)*** The following groups have been defined:

Group “Super Early” (SE): laparotomy < 60 min

Group “Early” (E): laparotomy 60–180 min

A 60-minute cut-off was selected prior to the data collection started, as the study as this time window allows for realistic inclusion of efficient trauma diagnostics and preparation times for surgery. Furthermore, we aim to finish the diagnostic work-up in the trauma bay within 30 min and transfer to the operating room, ICU or trauma ward should be completed within 60 min. Furthermore, the set goals for trauma care in our region are partly extracted from the concepts related to the `golden hour of shock`. Applying this cut-off value for the current study prevents making the analysis overcomplicated. A different cut-off point than 60 min would be harder to explain. One hour is a realistic, simple and logical cut-off point based on the experiences of the involved authors. ***(Correction 1.3)***

### Indications for emergency surgery

According to our guidelines, non-surgical management is recommended for hemodynamically stable BAT patients without evidence of hollow viscus injury [13]. Indications for emergency laparotomy for BATs include hypotension and positive FAST/CT or clinical evidence of intra-abdominal bleeding, peritonitis, free or retroperitoneal air, and rupture of the hemidiaphragm, rupture of the gastrointestinal tract or intraperitoneal bladder injury [14].

Non-operative management (NOM) at our institution includes initial observation in the intensive care unit (ICU) or intermediate care unit (IMC), frequent vital signs and physical examinations (including abdominal and neurological examinations), administration of fluids, and serial measurements of routine laboratory values [13]. A contrast-enhanced CT scan is usually performed before admission to the IMC/ICU.

The goal of intravenous fluid therapy in all patients without relevant traumatic brain injury (TBI) is to maintain a systolic blood pressure of at least 90 mmHg [15]. In our trauma centre, angioembolisation is considered in patients initially selected for NOM whose haemodynamic status continues to deteriorate due to ongoing splenic haemorrhage [16]. Treatment guidelines and selection criteria for non-operative management, surgical intervention, and angioembolisation remained unchanged throughout the study period. This was further supported by the absence of any significant changes in leadership of the department or trauma bay resulting in radical shifts in utilized guidelines relevant to our treatment strategies. ***(Correction 1.4a)***

### Outcome parameters and statistics

Outcomes were assessed by comparing the length of stay (LOS), duration of intensive care and/or ventilation, survival time, re-look surgeries and mortality. In terms of data representation, continuous data were presented as mean and standard deviation respective median and IQR and categorical variables were presented as numbers and percentages. Statistical analysis and visualisation were performed in R using the ‘Stat’ and ‘Tableone’ packages. Microsoft Excel was used for data visualization. Data were visually assessed for normality using histograms. Categorical binary outcome data were analysed using a one-tailed Fisher’s exact test, while demographic data were assessed with a two-tailed Fisher’s exact test. An adjusted linear model was calculated to identify independent risk factors for mortality. Non-binary categorical data were assessed using the chi-squared test with Yates’ correction for continuity. Additional propensity score–matching analyses were performed as well. The significance level was set at 0.05.

## Results

### Patient and trauma characteristics

**Fig. 1:**
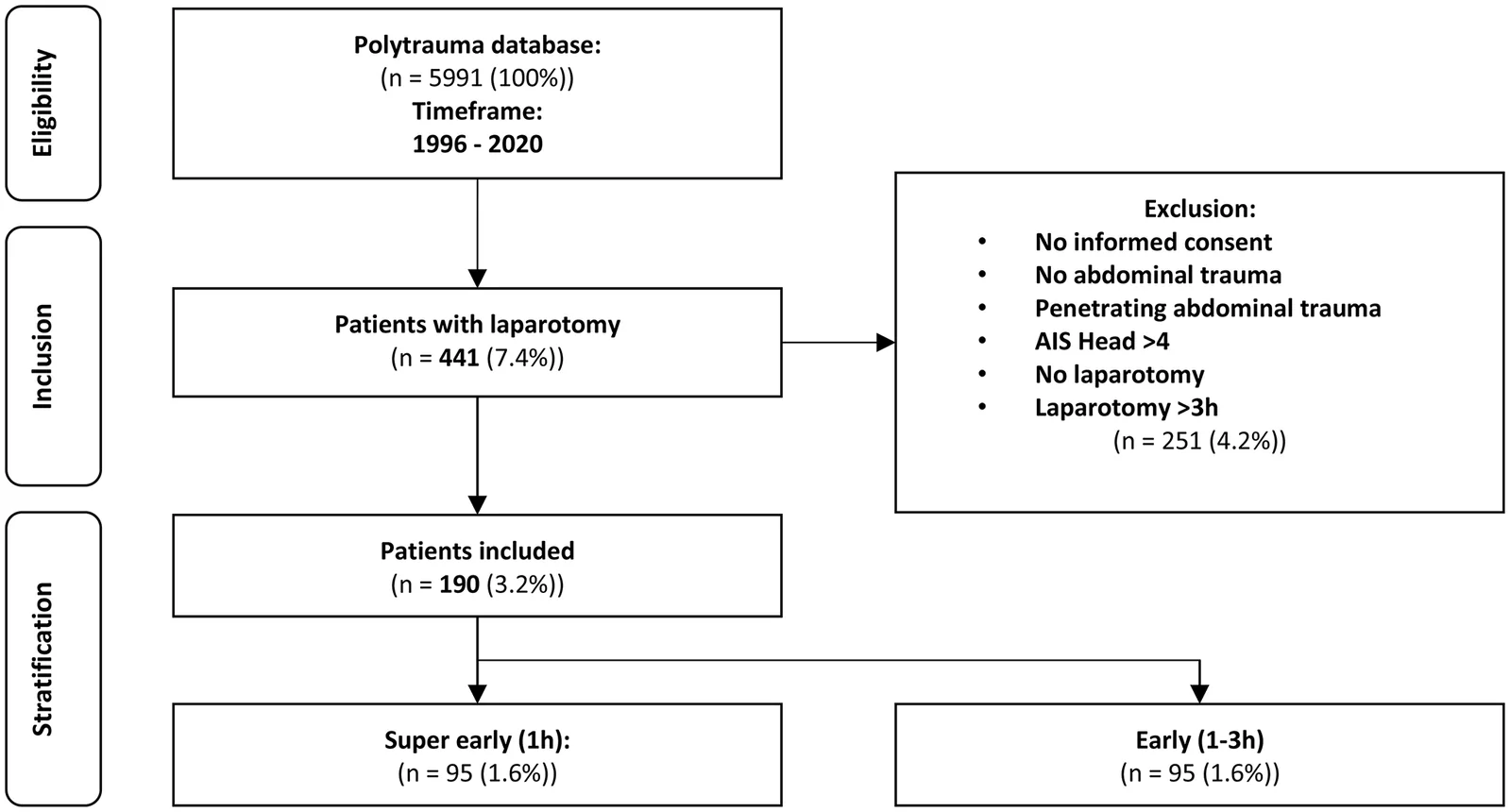
Flowchart of patient inclusion process

A total of 441 patients with BAT underwent a laparotomy during the study period (Fig. 1), of which 190 met the inclusion criteria. The age at the time of hospitalisation was comparable between the groups, with a mean age of 42.17 (± 17.4) years in the “Super Early” (SE) versus 42.45 (± 16.75) in the “Early” (E) group. The majority of the patients in both groups were male (SE: 69.5% vs. E: 75.8%, *p* = 0.416). The “Super Early” group exhibited a significantly higher median (interquartile range) ISS (41 (16.75) vs. 34 (16), *p* = 0.01) and BMI (SE: 26.60 ± 4.07 vs.

E: 24.26±5.81, *p*=0.014). No differences in trauma mechanism, gender distribution and age were encountered between groups. ***(Correction 1.16)*** The most common trauma mechanism was road traffic accidents, with a prevalence of 65.35% in the “Super Early” group and 63.2% in the “Early” group. The overall injury mechanisms were comparable between the groups (*p* = 0.943).

The 95 patients included in the “Super Early” group underwent surgery within 60 min of admission, with a mean time to laparotomy of 36.33 min (± 18.97). In contrast, the patients in the “Early” group received a laparotomy 60 to 180 min after admission (mean time to laparotomy: 106.34 ± 32.69 min) (Table 1).

**Table 1:** Patient and trauma characteristics as well as time to laparotomy after trauma bay admission. ***(Correction 1.16)***

	Super Early	Early	*p*-Value
n	95	95	
Age, mean (± SD)	42.14 (± 17.45)	42.45 (± 16.75)	0.899
Female Sex, n (%)	29 (30.5)	23 (24.2)	0.416
BMI, mean (± SD)	26.60 (± 4.07)	24.26 (± 5.81)	0.014
ISS, median (IQR)	41 (17)	34 (16)	0.01
Trauma mechanism, n (%)			0.943
Home accident	3 (3.2)	6 (6.3)	
Self-endangerment	11 (11.6)	11 (11.6)	
Sport accident	3 (3.2)	2 (2.1)	
Traffic accident	62 (65.3)	60 (63.2)	
Unclear	1 (1.1)	1 (1.1)	
Violent crime	0 (0.0)	1 (1.1)	
Workplace accident	15 (15.8)	14 (14.7)	
Admission to laparotomy (min), mean (± SD)	36.33 ± 18.97	106.34 ± 32.69	< 0.001

There were no significant differences between the AIS head (Median (IQR) = SE: 1(3) vs. E: 1(3), *p* = 0.713), thorax (SE: 3(1.75) vs. E: 3(2.5), *p* = 0.128), abdomen (SE: 4(2) vs. E: 4(1.5), *p* = 0.701) and extremities (SE: 3(2) vs. E; 2(1), *p* = 0.232).

The patients in the “Super Early” group exhibited a significantly lower systolic blood pressure (SE: 104 ± 34.24 vs. E: 118 ± 27.5 mmHg, *p* = 0.004), temperature (SE: 34.27 °C vs. E: 35.10, *p* = 0.002), GCS (7.10 vs. 9.65), haemoglobin (SE: 74.40 ± 32.79 vs. E: 98.41 ± 33.02, *p* < 0.001), hematocrit (SE: 21.62 ± 10.42 vs. E: 27.66 ± 11.46, *p* < 0.001), and higher lactate (SE: 6.01 ± 3.70 vs. E: 3.96 ± 2.96, *p* < 0.001). The heart rate was comparable between both groups (SE: 107.14 ± 27.56 vs. E: 100.92 ± 22.22, *p* = 0.125) (Table 2).

**Table 2:** Injury specific and physiological parameters at admission

	Super Early	Early	*p*-Value
AIS Head, median (IQR)	1 (3)	1 (3)	0.713
AIS Thorax, median (IQR)	3 (1.75)	3 (2.5)	0.128
AIS Abdomen, median (IQR)	4 (2)	4 (1.5)	0.701
AIS Extremities, median (IQR)	3 (2)	2 (1)	0.232
Clinical parameters on admission			
Systolic blood pressure, mean (± SD)	104.14 (± 34.24)	118.82 (± 27.50)	0.004
Heart frequency, mean (± SD)	107.14 (± 27.56)	100.92 (± 22.22)	0.125
Temperature (Celsius), mean (± SD)	34.27 (± 1.68)	35.10 (± 1.47)	0.002
GCS, mean (± SD)	7.10 (± 5.25)	9.65 (± 5.48)	0.001
Lactate, mean (± SD)	6.01 (± 3.70)	3.96 (± 2.96)	< 0.001
Hematocrit, mean (± SD)	21.62 (± 10.42)	27.66 (± 11.46)	< 0.001
Hemoglobin, mean (± SD)	74.40 (± 32.79)	98.41 (± 33.02)	< 0.001

Cumulatively, the most common trauma pattern observed was solid organ injury (SE: *n* = 56 (58.9%) vs. E: *n* = 51 (53.7%), *p* = 0.559), with the spleen being most frequently affected (SE: *n* = 32 (33.7%) vs. E: *n* = 31 (32.6%), *p* = 1). The liver was significantly more frequently injured (*p* = 0.008) in the “Super Early” group (*n* = 29 (30.5%)) than in the “Early” group (*n* = 13 (13.7%)). There was no significant difference in the incidence of injuries to the urogenital tract (kidneys, urethra, uterus) and the pancreas (SE: *n* = 13 (13.7%) vs. E: *n* = 20 (21.1%), *p* = 0.250). Hollow organ injuries occurred in roughly every fourth patient (SE: *n* = 23 (24.2%) vs. E: *n* = 24 (25.3%), *p* = 1). Serous membrane lesions, including those affecting the serosa, mesenterium and peritoneum lesions were observed with greater frequency in the “Super Early” group (SE: *n* = 18 (18.9%) vs. E: *n* = 7 (7.4%), *p* = 0.030). The number of major vascular lesions was comparable between the groups (SE: *n* = 12 (12.6%) vs. E: *n* = 6 (6.3%), *p* = 0.215), as well as other injury patterns such as diffuse bleeding, ruptured hemidiaphragm or abdominal compartment (SE: *n* = 17 (17.9%) vs. E: *n* = 23 (24.2%), *p* = 0.374). Multiple patients presented with a combination of the aforementioned intra-abdominal injury patterns (Table 3).

**Table 3:** Specific intra-abdominal injury pattern

Injury pattern	Super Early	Early	*p*-Value
Solid organ injury, n (%)	56 (58.9)	51 (53.7)	0.559
Spleen, n (%)	32 (33.7)	31 (32.6)	1
Liver, n (%)	29 (30.5)	13 (13.7)	0.008
Urogenital tract/pancreas, n (%)	13 (13.7)	20 (21.1)	0.250
Hollow organ injury, n (%)	23 (24.2)	24 (25.3)	1
Serous membranes, n (%)	18 (18.9)	7 (7.4)	0.030
Major vascular injury, n (%)	12 (12.6)	6 (6.3)	0.215
Other, n (%)	17 (17.9)	23 (24.2)	0.374

### Outcome parameters

Our primary outcome, mortality, was significantly higher in the “Super Early” group (SE: *n* = 49 (51.6%) vs. E: *n* = 25 (26.3%), *p* < 0.001).

There were no significant differences in LOS (days) (SE: 24.01 ± 28.84 vs. E: 26.26 ± 24.32 days, *p* = 0.561), LOS ICU (days) (SE: 12.61 ± 18.78 vs. E: 11.74 ± 74, *p* = 0.707), ventilation days (SE: 8.68 ± 14.28 vs. E: 7.15 ± 8.05, *p* = 0.365) as well as the amount of re-look operations (SE: 0.61 ± 1.36 vs. E: 1.11 ± 2.55) between groups (Table 4).

**Table 4:** Outcome parameters

	Super Early	Early	*p*-Value
LOS days, mean (± SD)	24.01 (± 28.84)	26.26 (± 24.32)	0.561
LOS ICU days, mean (± SD)	12.61 (± 18.78)	11.74 (± 12.09)	0.707
Ventilation days, mean (± SD)	8.68 (± 14.28)	7.15 (± 8.05)	0.365
Re-look operations, mean (± SD)	0.61 (± 1.36)	1.11 (± 2.55)	0.097
Mortality, n (%)	49 (51.6)	25 (26.3)	< 0.001
Survival time (days), mean (± SD)	4.47 (± 15.99)	3.88 (± 6.44)	0.823

**Fig. 2:**
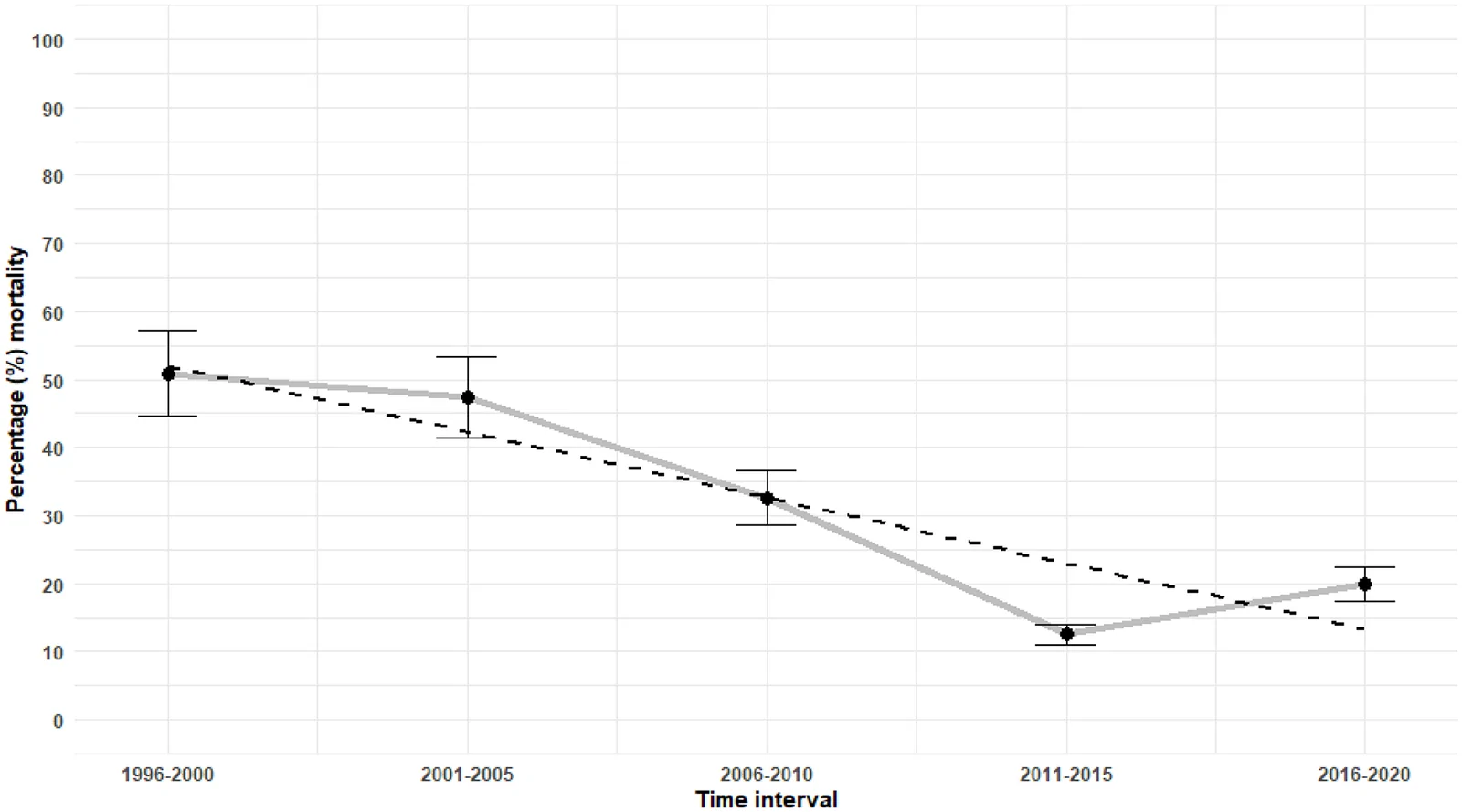
Mortality rate over time; displayed as mean with 95% CI. Dashed-line = Overall trend

Death due to hemorrhagic shock was significantly more common in the “Super Early” group (SE: *n* = 35 (36.8%) vs. E: *n* = 13 (13.7%), *p* < 0.001). There were no significant differences in mortality due to TBI (SE: *n* = 4 (4.2%) vs. E: *n* = 2 (2.1%), *p* = 0.682), organ failure / sepsis (SE: *n* = 6 (6.3%) vs. E: *n* = 8 (8.4%), *p* = 0.782) or other causes (SE: *n* = 5 (5.3%) vs. E: *n* = 2 (2.1%), *p* = 0.444) (Table 5). Overall mortality decreased over the course of the study period from 1996 to 2020 (Fig. 2).

**Table 5:** Causes of death

Cause of death	Super Early	Early	*p*-Value
TBI, n (%)	4 (4.2)	2 (2.1)	0.682
Hemorrhagic shock, n (%)	35 (36.8)	13 (13.7)	< 0.001
Organ failure / sepsis, n (%)	6 (6.3)	8 (8.4)	0.782
Other, n (%)	5 (5.3)	2 (2.1)	0.444

### Multivariate analysis

**Fig. 3:**
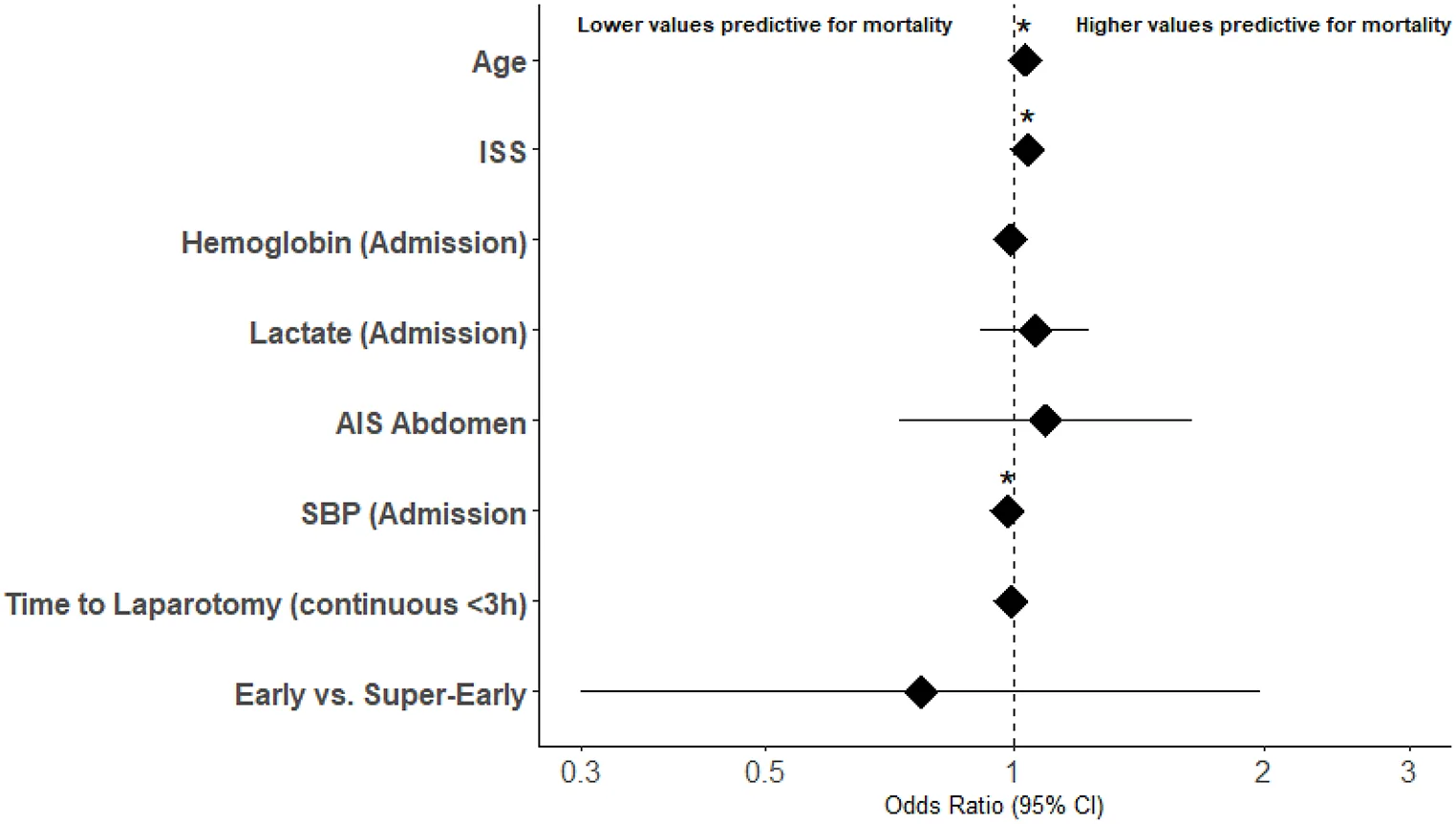
Forest-Plot presenting independent predictive qualities of selected parameters of interest

An adjusted logistic regression for mortality yielded a higher age (OR 1.03 CI: 1-1.06, p = 0.037), higher ISS (OR 1.04 CI: 1-1.08, p = 0.049) and lower systolic blood pressure (OR 0.981 CI: 0.96–0.99, p = 0.024) as independent predictors for mortality. Further factors investigated, such as group affiliation (“Super Early”/”Early”), as well as absolute time to operation within 3 h, AIS of the abdomen, hemoglobin and lactate, yielded no significant predictive ability (Table 6; Fig. 3). In addition, in order to adjust by an alternative method for potential selection bias, an additional propensity matching analysis has been performed. The results are provided in Supplementary Data File 1, as the outcome is in line with the results extracted from the multivariable analysis. ***(Correction 1.7)***

**Table 6:** Independent predictors for mortality (adjusted)

Parameter	OR	Lower CI	Upper CI	*p*-Value
Group (Early / Super Early)	0.772	0.301	1.98	0.592
Time to OP	0.993	0.982	1	0.25
Age	1.03	1	1.06	**0.037**
ISS	1.04	1	1.08	**0.049**
AIS Abdomen	1.09	0.728	1.64	0.668
Hemoglobin Admission	0.989	0.973	1.01	0.180
Lactate Admission	1.06	0.909	1.23	0.496
Systolic blood pressure	0.981	0.964	0.998	**0.025**

## Discussion

The key results of this study may be summarised as follows:

1. An emergency laparotomy in polytrauma patients with BAT is associated with a high mortality rate.
2. Time to emergency laparotomy in this cohort was not an independent predictor of mortality, and super early surgery, defined as laparotomy within 60 min, is not associated with a better outcome than later laparotomy. ***Correction 1.1***.
3. A higher age, higher ISS and lower SBP on admission are independent predictors of a higher mortality after BAT.

Our findings suggest that a short, clinically justified delay may not negatively affect mortality in all patients requiring emergency laparotomy after trauma. In selected patients and as part of pre-optimization prior to emergency surgery, this time window can be utilized to perform additional diagnostics such as contrast-enhanced CT imaging. Especially when the additional imaging is likely to support surgical decision-making [17]. However, it is paramount to view this in the context of the patient’s physiology, as older patients with a higher ISS and lower SBP on admission are especially at risk of a poor outcome. In most cases, it is not advisable to delay emergency laparotomy for the sake of better diagnostics. ***Correction 1.9***.

The relatively high mortality rate and length of ICU and hospital stay in this study can be explained by the high severity of the trauma in the cohort, as reflected by an ISS of 41 (SE) and 34 (E), respectively, with an admission lactate of 6 (SE) and 4 (E). The majority of the patients with a fatal outcome were in the “Super Early” group, and the most common cause of death was haemorrhagic shock, which aligns with the findings in existing literature [18]. Previous research reports an immediate mortality rate of up to 21% and a 1–2 year mortality of around 23–30%, [18–20] which is comparable to this study. (Fig. 2). The relatively high ISS score in our cohort is most likely due to the excellent pre-hospital care system and very short transport times [21]. Consequently, a relatively high proportion of patients with severe injuries often arrive at hospital in a pre-lethal condition.

Unarguably, immediate haemorrhage control should be of utmost priority in hemodynamically unstable patients, as recommended even more clearly in the most recent editions of trauma guidelines, including Advanced Trauma Life Support [22]. Although the current study does demonstrate that there is no difference in survival between matched patients who undergo laparotomy within 60 min and those who undergo laparotomy between 60 and 180 min. This makes it tempting to hypothesise that a subset of patients requiring emergency laparotomy is not at increased risk of mortality when surgery is delayed by a short time. This provides a window of opportunity to pre-interventionally optimise the physiology of the patient (e.g. hypothermia, coagulopathy or treatment of non-abdominal blood loss) or to perform additional diagnostics. Further research is needed to precisely identify the characteristics of this specific subset of trauma patients. ***(Correction 1.10)***

Interestingly, it appears that traditional laboratory parameters of impaired physiology (e.g. hemoglobin, lactate) are not independent predictors of increased mortality in this patient cohort. This finding may be related to the widespread implementation of restrictive fluid therapy in the pre-hospital setting, combined with the short rescue time in our region [21, 23]. Alternatively, this interesting finding may be related to an underpowered analysis, or there may be unidentified confounding factors involved. ***(Correction 1.11)***

Previous research offers very limited data specific to the impact of timing of emergency laparotomy in BAT especially, in the first few hours post-insult. A large multi-centre analysis of the Israeli Trauma Registry found that patients with BAT and ISS > 16 and GCS < 15 had a significantly higher mortality if they underwent immediate surgery (within 60 min) in comparison to the expedient group (surgery within 60–120 min). However, it has to be taken into account that this study was limited to hemodynamically unstable patients (defined by SPB < 90 on admission) who had an isolated abdominal injury or less severe concomitant injuries (AIS 1–2 only). Additionally, this study did not perform a multivariate analysis in order to help identify independent predictors for mortality [24].

Remick et al. demonstrated that early emergency surgery following trauma is generally associated with improved survival outcomes, particularly in cases of penetrating trauma or prolonged haemodynamic instability [11]. However, it is important to note that the study analysed a highly heterogeneous group of trauma patients who received a range of surgical interventions and only included cases with a fatal outcome within 4 h of injury. Interestingly, there was no significant difference in time to death between hypotensive and normotensive patients requiring laparotomy. Furthermore, a systematic review by Harmsen et al. demonstrated that prehospital time influenced outcome only in hemodynamically unstable patients with penetrating injuries, but not in hemodynamically stable trauma patients [25].

A multicenter study was conducted by Watts et al. on 2632 patients with a hollow viscus injury [10]. The study demonstrated that a delay in operative treatment of more than 24 h led to a significant increase in mortality (5% vs. 16%) only if an isolated small bowel perforation had occurred. However, it is important to note that the cohort of patients differed significantly from ours in terms of the overall injury pattern and severity. Furthermore, no specific information was provided on what early interventions (within three hours) were performed. Additional data on the effect of delayed intervention in blunt abdominal trauma is available and broadly aligns with the findings by Watts et al. [26–28]. Nevertheless, none of the above addressed the impact of relatively prompt trauma laparotomy (within 3 h of admission) specifically, but rather a more delayed intervention.

### Limitations of the study

This study focuses on a specific cohort of severely injured polytrauma patients. It is not applicable to isolated blunt abdominal trauma, as it is influenced by additional injury patterns. Furthermore, only patients with BAT were studied, and findings of this investigation should not be extrapolated to patients with penetrating trauma, as penetrating trauma leads to different injury patterns and, specifically, higher rates of concurrent hollow viscus injuries [9].

Timing of surgery was calculated based upon arrival to the trauma bay, as the exact time of the injury could not be determined retrospectively.

For the purposes of this study, we have only included patients who received emergency laparotomy within three hours. Therefore, the results cannot be applied to other timeframes. Furthermore, the dataset covers a 25-year period. Improved resuscitation and emergency procedures over time have contributed to the overall improvement in mortality rate, and an overview of altered mortality during the study has been provided as Supplementary Data File 2. As this was not the scope of this research project, we did not identify trends that resulted in the improved mortality over time. The increased use of tranexamic acid, ROTEM studies and blood component therapy in the trauma bay, alongside restricted volume resuscitation, and improved pre-hospital trauma care, may also be contributing factors [23, 29–31]. **(Correction 1.5)** However, it is important to note that the indications (including the thresholds) for interventions, as well as the criteria for admission to the trauma bay, remained unchanged during the study period. As the authors assume that both groups (operated within and after 60 min) benefited from these improvements mortality over time, a selection bias due to asymmetric benefit of both study conditions over time is unlikely. ***(Correction 1.4b)***

In addition, due to the design of the study, selection biases may be involved, as patients undergoing laparotomy within one hour had higher ISS than their counterparts operated on later. The exact reason for this delay (e.g. logistics, additional imaging, multi-source bleeding) is currently unclear. Additional propensity matching (Supplementary Data File 1) has been performed to further correct for group differences in the raw data analysis. ***(Correction 1.13****)* Finally, a relatively large dataset was available, including parameters describing patient and trauma characteristics, treatment strategies, and outcomes. However, additional parameters of potential interest (including pre-hospital data) would include alternative laboratory markers such as base excess and transaminases, transfusion requirements, duration of surgery, body temperature, and more detailed coagulation parameters. ***(Correction 1.6)***

## Conclusion

The current study is the first to study to focus on the impact of time to emergency laparotomy for blunt abdominal trauma on outcome in detail. It was demonstrated that time to emergency laparotomy was not an independent predictor of mortality when performed within 180 min after admission. More specifically, super-early laparotomy performed within 60 min was not associated with better outcomes than laparotomy performed later. Higher trauma load (ISS), older age, and impaired haemodynamics, reflected by lower systolic blood pressure on admission, were independent predictors of mortality after blunt abdominal trauma. These findings should be interpreted cautiously given the retrospective design and not be taken to imply that delays to emergency laparotomy should routinely be tolerated. Rather, they highlight the importance of further evaluating how timely surgical intervention can be balanced with selected pre-operative investigations in appropriately selected patients. Future multicentre prospective studies should aim to identify patient subgroups in whom a short delay to laparotomy may allow more comprehensive diagnostics or pre-operative optimization without compromising outcomes.* (****Correction 1.14/1.15)***.

## Supplementary Information

Below is the link to the electronic supplementary material.

Supplementary Material 1

## Data Availability

The data that support the findings of this study are not openly available due to reasons of sensitivity but are available from the corresponding author upon reasonable request.

## Abbreviations

BAT: Blunt abdominal trauma
AIS: Abbreviated injury score
CT: Computer tomography
DPL: Diagnostic peritoneal lavage
E: Early
ISS: Injury severity score
SE: Super Early
BMI: Body mass index
NOM: Nonoperative management
ICU: Intensive care unit
IMC: Intermediate care unit
TBI: Traumatic brain injury
