# Supplementary material for: Emergency surgery for blunt abdominal trauma in polytrauma – the impact of time to laparotomy on outcomes
Source: Eur J Trauma Emerg Surg. 2026 Oct 19;52(1):322. doi: 10.1007/s00068-026-03332-z (PMC13680524; doi:10.1007/s00068-026-03332-z)
Supplement: Supplementary file 1 [file 68_2026_3332_MOESM1_ESM.docx]

Supplementary data file 1: **Results of caliper propensity score matching analysis**

***METHODS:*** to account for confounding by indication, propensity score matching was performed using a nearest-neighbor approach with a caliper of 0.2. The propensity score was estimated based on baseline characteristics, including age, injury severity (ISS), abdominal injury severity, hemoglobin, lactate, and systolic blood pressure at admission. Balance between groups was assessed using standardized mean differences.

**Table S1.1**

|  | **Early (n = 42)** | **Super Early (n = 42)** | **SMD** |
| --- | --- | --- | --- |
| **Baseline characteristics** |  |  |  |
| Age, years (mean ± SD) | 40.76 ± 16.91 | 40.45 ± 14.87 | 0.019 |
| ISS (mean ± SD) | 37.07 ± 13.30 | 36.17 ± 14.69 | 0.065 |
| AIS Abdomen (mean ± SD) | 3.67 ± 1.48 | 3.67 ± 1.41 | <0.001 |
| Hemoglobin (g/L) (mean ± SD) | 91.76 ± 29.43 | 96.76 ± 33.21 | 0.159 |
| Lactate (mmol/L) (mean ± SD) | 4.16 ± 2.33 | 4.16 ± 3.18 | 0.001 |
| Systolic BP (mmHg) (mean ± SD) | 114.02 ± 29.58 | 115.74 ± 30.84 | 0.057 |
|  |  |  |  |
| **Outcome** |  |  |  |
| Mortality, n (%) | 13 (30.9%) | 11 (26.2%) | – |
| Odds ratio (95% CI) | - | 0.79 (0.30–2.05) | p = 0.63 |

Table S1.1: Baseline characteristics and outcomes of the propensity score matched cohort. Propensity score matching was performed using nearest-neighbor matching with a caliper of 0.2. Data are presented as mean ± standard deviation or number (percentage). Balance between groups was assessed using standardized mean differences (SMD), with values <0.1 indicating adequate balance. Mortality was compared between groups using logistic regression in the matched cohort.

**Figure S1.1**

**Figure S1.2**

Supplementary Data File 2: **Mortality over time**

***METHODS****:* To evaluate the impact of calendar time on mortality, a multivariable logistic regression model was constructed with calendar year entered as a continuous variable. The analysis was adjusted for age, injury severity (ISS), and physiological parameters at admission, including lactate, systolic blood pressure, and Glasgow Coma Scale.

**Table S2.1**

| **Factor** | **OR (95% CI)** | **p-value** | **Interpretation** |
| --- | --- | --- | --- |
| Calendar year (per year) | 0.92 (0.84–0.99) | 0.030 | ~8% decrease in mortality per year |
|  |  |  |  |
| **Adjustment variables** | – | – | Age, ISS, lactate, systolic blood pressure, and GCS were included in the model |

Table X: Adjusted association between calendar year and mortality derived from a multivariable logistic regression model (adjusted for age, ISS, lactate, blood pressure, and GCS).

**Figure S2.1**

Figure 2: Adjusted mortality over time based on multivariable logistic regression. The model was adjusted for age, injury severity (ISS), and physiological parameters at admission (lactate, systolic blood pressure, and Glasgow Coma Scale). Shaded areas represent 95% confidence intervals
